# Platelet-rich plasma vs corticosteroids for elbow epicondylitis: A systematic review and meta-analysis

**DOI:** 10.1097/MD.0000000000018358

**Published:** 2019-12-20

**Authors:** Ang Li, Hongbo Wang, Zhenghong Yu, Guangquan Zhang, Shiqing Feng, Liyun Liu, Yanzheng Gao

**Affiliations:** a Department of Orthopedics, Henan Provincial People's Hospital, People's Hospital of Zhengzhou University; b Henan University People's Hospital, Zhengzhou, Henan; c Department of Orthopedics, Tianjin Medical University General Hospital, Tianjin, China.

**Keywords:** corticosteroids, epicondylitis, meta-analysis, platelet-rich plasma

## Abstract

**Objective:**

The aim of this meta-analysis was to compare the effectiveness of platelet-rich plasma (PRP) vs corticosteroids for treatment of patients with lateral elbow epicondylitis.

**Methods:**

A literature search was performed in EMBASE, Medline, the Cochrane Library and PubMed. Randomized controlled studies comparing PRP with corticosteroids for the treatment of epicondylitis were included. The Cochrane Collaboration's tool for assessing the risk of bias was used to evaluate the methodological quality of the included trials. The Cochrane Collaboration's Review Manager software was used to perform the meta-analyses. The overall effect size of each anesthetic was calculated as the weighted average of the inverse variance of the study-specific estimates.

**Results:**

Seven randomized controlled trials were included in this review. The data from 2 studies were unavailable for meta-analysis, and the systematic review criteria were just achieved. Local corticosteroid injection yielded a significantly superior Disabilities of the Arm, Shoulder and Hand (DASH) score at 4 weeks (WMD, 11.90; 95% CI: 7.72 to 16.08; *P* < .00001; heterogeneity, χ^2^ = 0, I^2^ = 0%, *P* = 1.00) and 8 weeks (WMD, 6.29; 95% CI: 2.98 to 9.60; *P* = .0002, χ^2^ = 0, I^2^ = 0%, *P* = 1.00). Otherwise, it was noteworthy that a significantly lower VAS score (WMD, −2.61; 95% CI: −5.18 to −0.04; *P* = .05; heterogeneity, χ^2^ = 29.85, I^2^ = 97%, *P* < .00001) and DASH score (WMD, −7.73; 95% CI: −9.99 to −5.46; *P* < .00001, χ^2^ = 0.20, I^2^ = 0%, *P* = .66) existed in the PRP regimen than in the steroid regimen at the 24-week follow-up. More effective treatments were achieved in the PRP-treated patients than in the patients treated with corticosteroids (WMD, 3.33; 95% CI: 1.81 to 6.14; *P* = .000; heterogeneity, χ^2^ = 0.43, I^2^ = 0%, *P* = .51).

**Conclusions:**

Local corticosteroid injections demonstrated favorable outcomes compared with those of local PRP treatments for lateral elbow epicondylitis during the short-term follow-up period (4 weeks and 8 weeks post-treatment). Otherwise, at the long-term follow-up (24 weeks post-treatment), PRP injections had improved pain and function more effectively than corticosteroid injections.

## 1 Introduction

Lateral epicondylitis, commonly known as tennis elbow, is one of the most common soft tissue injuries in adults, mainly in the age range of 35 to 55 years.^[1]^ The typical symptoms include lateral elbow pain, pain with wrist extension, and weakened grip strength.^[2]^ Presently, the medications available for injection therapy included botulinum toxin A, autologous blood, glycosaminoglycan polysulfate, sodium hyaluronate, polidocanol, epinephrine, dextrose, sodium morrhuate, PRP, and corticosteroids.^[3–6]^ Injections with corticosteroids have been used since the 1950s.^[7]^ Currently, the use of corticosteroid injections is considered by many practitioners to be safe, especially for trials in refractory cases of epicondylitis. Corticosteroids have been shown to relieve pain of neurogenic origin.^[8,9]^ PRP is defined as an autologous blood product with a concentration of platelets greater than that at baseline. PRP releases some substances (such as vascular endothelial growth factor and transforming growth factor-β) that promote tissue repair and influence the reactivity of vascular and other blood cells in angiogenesis and inflammation.^[10]^ Many reports on the use of PRP for lateral epicondylitis described superior results compared to the results of other treatments.^[2,11–13]^

Though the effectiveness of PRP treatment is internationally proven, conflict exists among orthopedic surgeons. Many studies have compared steroid injections with PRP treatment^[12–17]^; however, which is more effective is unknown. Accordingly, we conducted a meta-analysis to compare the efficacy of PRP injections with that of corticosteroids in patients with lateral epicondylitis.

## 2 Methods

### 2.1 Literature search

The meta-analysis was registered in PROSPERO. The registration number is CRD42019137360. The following electronic databases were independently and extensively searched by two investigators from their inception through August 2018: EMBASE, Medline, the Cochrane Library, and PubMed. The search keywords were centered on the terms “platelet-rich plasma”, “corticosteroid”, and “elbow epicondylitis”, which were adjusted to each database as necessary. In addition, the bibliographies of the included studies and dissertations were searched for additional publications. The search language was restricted to English.

### 2.2 Inclusion and exclusion criteria

PRISMA guidelines were followed for the inclusion of studies in the meta-analysis. The detailed description of the inclusion criteria as follows:

(1) trials had to be properly randomized;
(2) no additional agents or interventions confounded the comparison;
(3) the patients in the trials were given a bolus dose via local solution injection;
(4) with respect to trials with several intervention groups, the eligibility of each individual group was evaluated, and only those qualified were included. Case reports, editorials, experimental studies and commentaries were excluded.

### 2.3 Data collection

After duplicates were removed and the study selection process was completed, the titles and abstracts were scanned by 2 independent investigators. The relevant data were extracted following a predetermined standardized procedure and included the first author, the year of publication, the country, the demographic characteristics of the participants, and the treatment regimen for each group. All data were verified for internal consistency, and controversies were settled by consensus or discussion with a third author. When inadequate information existed in the studies, contacting the first authors to obtain and clarify the relevant data was essential, as specified by the standardized protocol.

### 2.4 Quality assessment

The Cochrane Collaboration's tool for assessing the risk of bias was used to evaluate the methodological quality of the included trials. This tool focuses on the internal validity of the trial and assessment of the risk of possible bias in different phases of trial conduction. The following items were assessed: random sequence generation, allocation concealment, blinding of participants, personnel and outcome assessment, incomplete outcome measures, selective outcome reporting and other types of bias. Each item was qualified as low risk (L), unclear risk (U), or high risk (H). All assessments were conducted by two reviewers, independent of each other. Controversies were settled by consensus or discussion with a third author.

### 2.5 Statistical analysis

The Cochrane Collaboration's Review Manager software (RevMan Version 5.2, The Cochrane Collaboration, Copenhagen, 2014) was used to perform the meta-analyses. The overall effect size of each anesthetic was calculated as the weighted average of the inverse variance of the study-specific estimates. For dichotomous variables, we listed individual and pooled statistics as odds ratios with 95% confidence intervals. For continuous time-to-union data, we pooled the weighted mean time-to-union with the associated 95% confidence interval and listed the individual means and standard deviations.^[18]^ Heterogeneity among the individual studies was evaluated based on Cochrane Q test and the I^2^ index, which express, as a percentage, the proportion of variability of the results due to heterogeneity as opposed to sampling error.^[19]^ Considerable heterogeneity was determined when the Cochrane's Q test resulted in *P* < .10 and I^2^ above 75%.^[20]^ In such cases, a random effect model was selected for analysis. Conversely, a fixed effect model was used.^[21]^ If essential, subgroup analysis was conducted to identify and explain the heterogeneity. A *P* value less than .05 was considered significant for all statistical tests.

### 2.6 Ethical statement

As all analyses were conducted on data from previously published studies; ethical approval was not necessary.

## 3 Results

### 3.1 Search results

**Figure 1:**
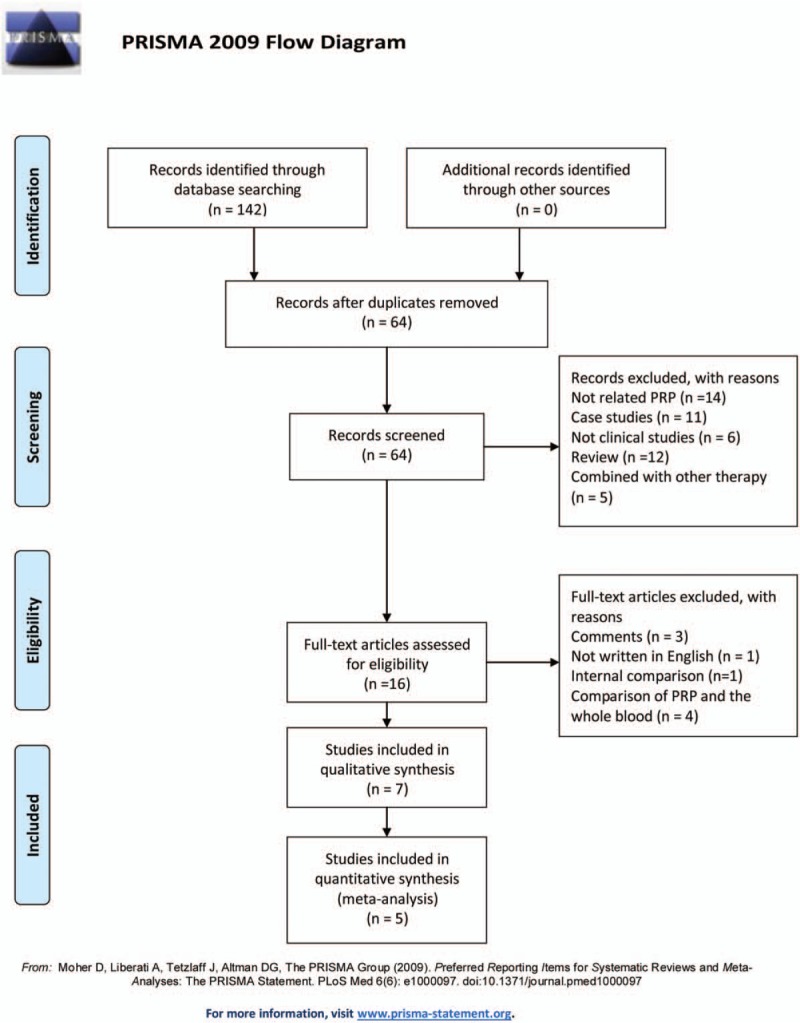
Flow diagram of study selection.

Figure 1 contains a flowchart that describes the process by which we screened and selected trials. The initial literature search yielded 142 articles in all. Duplicate checking and title and abstract screening resulted in 16 publications, and the full texts of all 16 articles were available. Among these, 3 were excluded because the articles were commentaries; 1 was excluded because the article was not written in English; 4 were excluded owing to comparing PRP and whole blood in the treatment of elbow epicondylitis; and 1 was excluded because the article was an internal comparison in the same patient. In addition, a manual search of relevant references did not identify any additional studies. Finally, 7 randomized controlled trials (RCTs)^[12–17,22]^ of intermediate to high quality were eligible for inclusion in this review.

### 3.2 Characteristics of the trials

Table 1 shows the detailed characteristics of the RCTs included. Of the 7 citations included, 2 citations^[13,14]^ were a series of articles from the same double-blind randomized controlled trial. The follow-up period was 2 years in the 2011 report.^[13]^ The follow-up period was 1 year in the 2010 report.^[14]^ Two studies^[13,14]^ were used as one for analysis in the meta-analysis. A pooled estimate was not conducted for the two reports. Data in the 2 studies^[12,22]^ were unavailable for meta-analysis, and the criteria for a systematic review was just achieved. Three trials were performed in India, 1 trial in Korea, 1 trial in Pakistan, and 1 trial with 2 reports in the Netherlands. A detailed production method for PRP was reported in 4 studies.^[13,14,16,17,22]^ Two trials^[12–14]^ were registered in the Clinical Trials database of the U.S. National Institutes of Health. The patients in 4 trials^[12–14,16,22]^ provided written informed consent before they underwent any study-related procedure. The appropriate sample size was calculated before the trials were conducted in 2 studies.^[12–14]^ Postinjection adverse events, such as fevers, rashes, local inflammation and exacerbation of pain, were reported in 3 trials.^[12,14,16]^ The mean duration of follow-up ranged from 2 to 52 weeks. One study^[12]^ contained 3 groups: a PRP group, a glucocorticoid group, and a placebo (saline) group. The placebo (saline) group was removed from the meta-analysis.

**Table 1:**
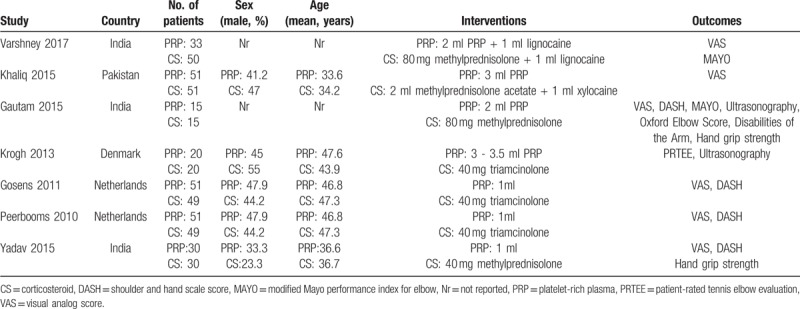
Summary of characteristics in the studies included.

The VAS score was reported in 4 trials.^[13,15–17]^ Two studies^[13,15]^ revealed that steroids were superior to PRP in terms of the VAS pain scores after a 4-week intervention (*P* < .01 and *P* = .023 respectively). Corticosteroids and PRP proved to be equally effective in the other 2 studies at the 4-week follow-up (*P* = .372 and *P* = .639, respectively). One study^[17]^ reported that the VAS score in the PRP group was higher than that in the corticosteroid group (*P* < .01) at the 8-week follow-up. Two studies revealed that no significant difference was detected after treatment for 8 weeks between the two arms (*P* = .249 and *P* = .411 for the 2 studies). Three studies^[13,16,17]^ found that PRP yielded significantly superior VAS scores after treatment for 24 weeks compared to the scores following treatment with local corticosteroid (*P* = .001, *P* = .0001 and *P* < .0001, respectively). Regarding the MAYO index, one RCT^[17]^ revealed that PRP treatment was superior to steroid treatment at 4 weeks post-treatment (*P* = .017). Two trials^[16,17]^ reported that corticosteroid and PRP proved to be equally effective at the 8-week follow-up (*P* = .578 and *P* = .471, respectively). Two trials^[16,17]^ revealed that the MAYO index was higher in the PRP group than in the corticosteroid group at the 24-week follow-up (*P* < .01 and *P* = .0001, respectively). The DASH score was available in 2 studies,^[13,17]^ and both studies found that a higher DASH score was achieved in the PRP group than in the steroid group at 4 weeks (*P* = .005 and *P* < .0001, respectively). One study^[13]^ revealed no significant difference between the 2 groups at 8 weeks (*P* = .06). One study^[17]^ reported that a higher DASH score was achieved in the PRP group (*P* = .003) than in the steroid group at 8 weeks. Two studies^[13,17]^ reported that no significant difference existed between the two arms at 12 weeks (*P* = .675 and *P* = .813 for the 2 studies, respectively).

### 3.3 Risk of bias assessment

Based on the Cochrane Collaboration's recommendation, blinding for the outcome assessment was reported in 2 studies.^[12,17]^ Three studies were randomized, double-blind, placebo-controlled trials.^[12–15]^ Randomization and comprehensive methodological processes were reported in 4 trials.^[12–16]^ The participants enrolled were consecutive patients in hospitals in 2 trials,^[13–15]^ and the selection bias could be high risk in these studies. The purposive, nonprobability sampling technique was used to enroll participants.^[15]^ The details of the risk of bias analysis are illustrated in Figure 5.

### 3.4 Visual analog score (VAS)

**Figure 2:**
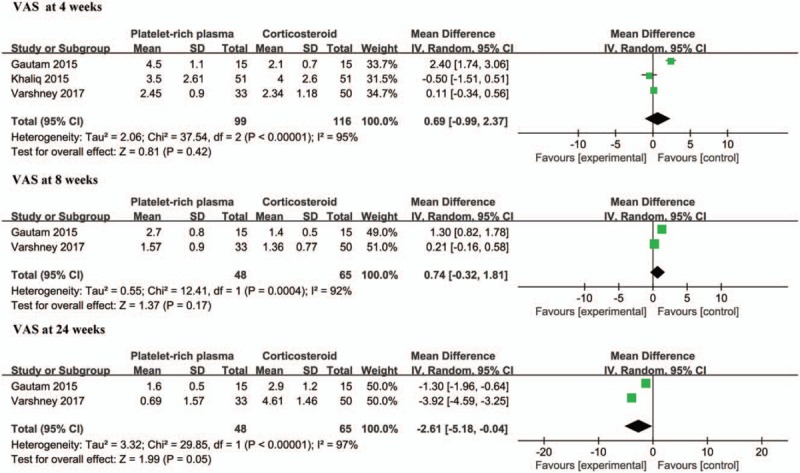
Forest plot for the VAS pain scores.

Pooled data found that no statistically significant difference was observed between the PRP group and the corticosteroid group in terms of the VAS scores after treatment for 4 weeks (WMD, 0.69; 95% CI: −0.99 to 2.37; *P* = .42). However, considerable heterogeneity was found (χ^2^ = 37.36, I^2^ = 95%, *P* < .00001). The random effect model was selected for the meta-analysis. Data on the VAS score after treatment for 8 weeks were available in 2 studies.^[16,17]^ Similarly, the meta-analysis revealed that no statistically significant difference existed between the PRP group and the corticosteroid group in terms of the VAS scores after treatment for 8 weeks (WMD, 0.74; 95% CI: - 0.32 to 1.81; *P* = .17). Additionally, considerable heterogeneity existed (χ^2^ = 12.41, I^2^ = 92%, *P* = .0004), and a random effect model was used. A pooled estimate found that the VAS scores after treatment for 24 weeks were higher in patients treated with corticosteroid than in PRP-treated patients (WMD, −2.61; 95% CI: −5.18 to −0.04; *P* = .05; heterogeneity, χ^2^ = 29.85, I^2^ = 97%, *P* < .00001) (Fig. 2).

### 3.5 Modified MAYO performance (MAYO) index for elbows

**Figure 3:**
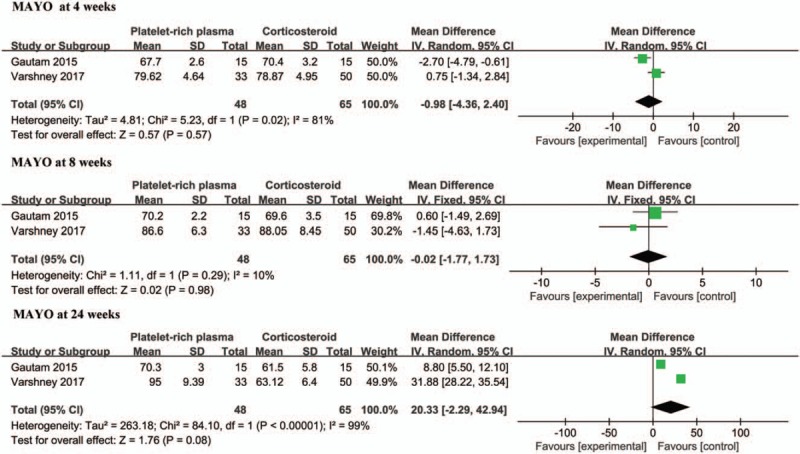
Forest plot for the MAYO index for elbows.

The MAYO index for elbows after treatment for 4 weeks was reported in 2 studies.^[16,17]^ Pooled data found that the MAYO index was almost comparable between the PRP group and the corticosteroid group (WMD = −0.98; 95% CI: −4.36 to 2.40, *P* = .57) and had considerable heterogeneity (χ^2^ = 5.23, I^2^ = 81%, *P* = .02). The meta-analysis demonstrated that local PRP injection did not yield a superior MAYO index for elbows compared to that of local steroid injection after an 8-week intervention (WMD, −0.02; 95% CI: −1.77 to 1.73; *P* = .98; heterogeneity, χ^2^ = 1.11, I^2^ = 10%, *P* = .29). The MAYO index for elbows at long-term follow-up (24 weeks) was reported in 2 trials.^[16,17]^ However, the meta-analysis showed that no statistically significant difference was observed between the PRP and corticosteroid groups at long-term follow-up (24 weeks) (WMD, 20.33; 95% CI: -2.29 to 42.94; *P* = .08). Considerable heterogeneity was found among the trial estimates (χ^2^ = 84.10, *P* < .0001), and the I^2^ index indicated that 99% of the variability across trials was due to heterogeneity rather than chance (Fig. 3).

### 3.6 Disabilities of the arm, shoulder and hand (DASH) score

**Figure 4:**
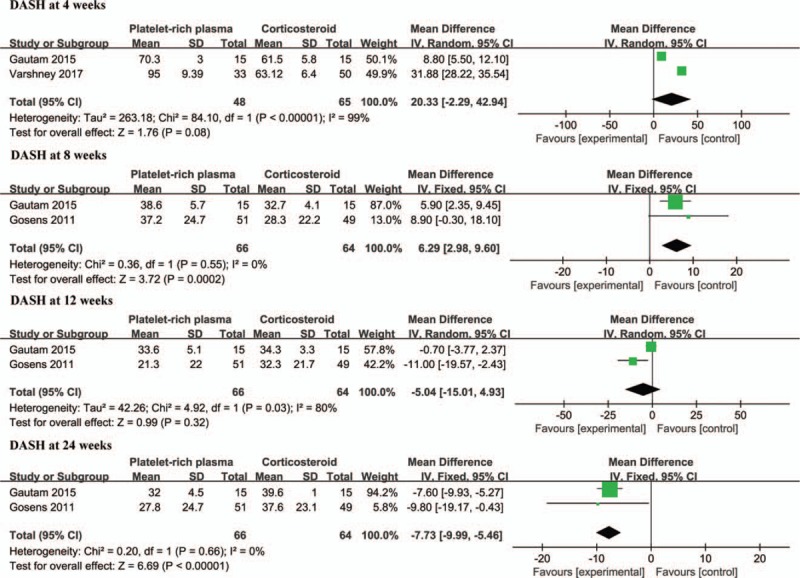
Forest plot for the DASH scores.

A pooled estimate revealed that a lower DASH score after treatment for 4 weeks was achieved in the corticosteroid group compared to that in PRP group (WMD, 11.90; 95% CI: 7.72 to 16.08; *P* < .00001; heterogeneity, χ^2^ = 0, I^2^ = 0%, *P* = 1.00). Additionally, it was noteworthy that corticosteroid injection yielded a significantly lower DASH score after treatment for 8 weeks compared to that in PRP group (WMD, 6.29; 95% CI: 2.98 to 9.60; *P* = .0002), with no heterogeneity existing (χ^2^ = 0, I^2^ = 0%, *P* = 1.00). The pooled data indicated that it was almost comparable between the 2 arms at the 12-week follow-up (WMD = −5.04; 95% CI: −15.01 to 4.93, *P* = .32). However, considerable heterogeneity was found (χ^2^ = 4.92, I^2^ = 80%, *P* = .03). The random effect model was used for meta-analysis. The meta-analysis demonstrated that a lower DASH score was achieved in the PRP group than in the steroid group at the 24-week follow-up (WMD, −7.73; 95% CI: −9.99 to −5.46; *P* < .00001), with no heterogeneity existing (χ^2^ = 0.20, I^2^ = 0%, *P* = .66) (Fig. 4). Two studies^[14,15]^ reported the effectiveness of the treatment or the successful treatment rate. The meta-analysis demonstrated that more effective treatments were achieved in PRP-treated patients than in patients treated with corticosteroids (WMD, 3.33; 95% CI: 1.81 to 6.14; *P* = .000; heterogeneity, χ^2^ = 0.43, I^2^ = 0%, *P* = .51) (Fig. 6).

**Figure 5:**
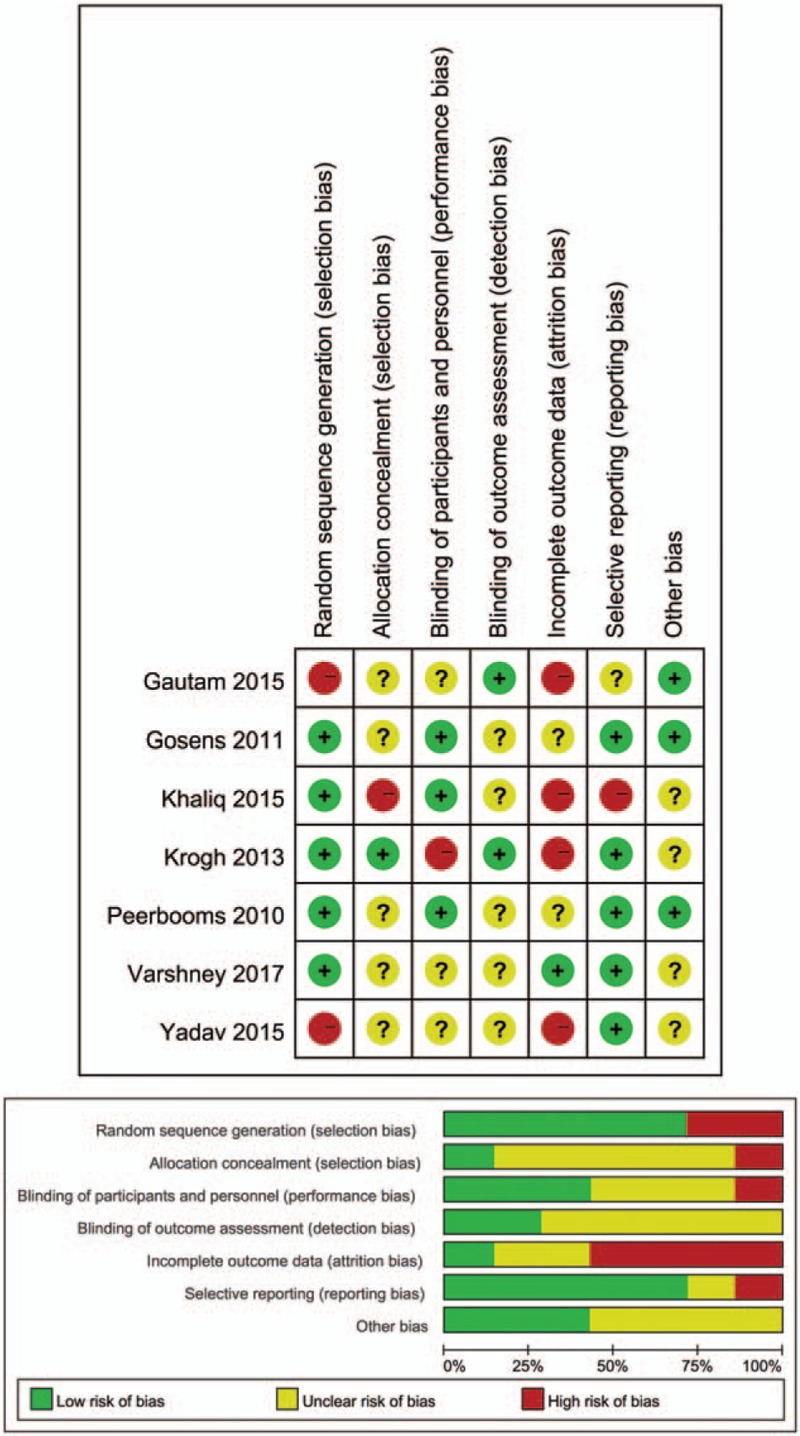
Risk of bias assessment of the included trials.

**Figure 6:**
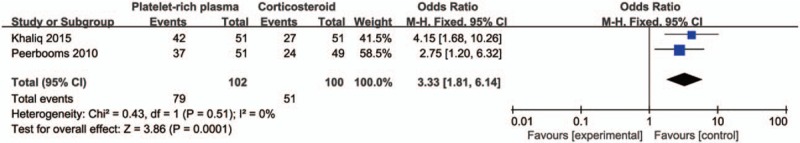
Forest plot for the effective treatment rates.

## 4 Discussion

The present meta-analysis showed that local corticosteroid injection yielded significantly lower DASH scores than PRP treatment during the short-term follow-up period (4 weeks and 8 weeks). Otherwise, it was noteworthy that significantly lower VAS and DASH scores existed for the PRP regimen than for the steroid treatment at the 24-week follow-up. Regarding the MAYO elbow index, no significant difference was established between the two arms during the whole follow-up period (4 weeks, 8 weeks and 24 weeks).

Overall, many clinical trials have compared the activity and efficacy of PRP vs corticosteroid injection in patients with elbow lateral epicondylitis.^[17,23]^ Local corticosteroid injection used to be the gold standard for the management of epicondylitis. However, it was reported that it had only a short-term effect (2–6 weeks).^[24–27]^ As an alternative treatment, several studies have shown that PRP injection had a long-term effect.^[11]^ In fact, the results of this meta-analysis support the above findings.

Lateral epicondylitis was initially believed to be an inflammatory process, but in 1979, it was described as the disorganization of normal collagen architecture by invading immature fibroblasts in association with an immature vascular reparative response.^[28–31]^ As a means of growth factor delivery, PRP has been used in maxillofacial and plastic surgery since the 1990s.^[32]^ The proposed release of growth factors, including interleukin-1β (IL-1β), epithelial growth factor (EGF), platelet-derived growth factor (PDGF), transforming growth factor-β (TGF-β), and vascular-endothelial growth factor (VEGF), could stimulate tenocyte proliferation and differentiation.^[33]^ Animal experiments showed that PRP could improve early neotendon properties so that the cells are able to perceive and respond to mechanical loading at an early time point, which may be responsible for the long-lasting effects of PRP treatment in epicondylitis.^[34]^

It has been suggested that the anti-inflammatory effect of corticosteroids is exerted by suppressing or dispersing the granulomatous response in traumatized tissue.^[35]^ Many studies noted short-term gains after steroid injection, with acute improvement in pain scores over the first 6 weeks but with no difference in pain by 3 months.^[26,27]^ When comparing corticosteroid injections with other conservative treatments or no treatment, no statistically significant or clinically relevant outcome measures favored corticosteroid injections at long-term follow-up.^[36]^ Based on this evidence, corticosteroids may aid in acute pain relief but not in the long-term treatment of lateral epicondylitis. The underlying mechanism is still unknown. Possible explanations could be related to the known short half-life of corticosteroids and a favorable natural history of epicondylitis. Furthermore, when patients become pain free, they resume the injurious activity without sufficient protective rehabilitation. Symptoms occur repeatedly with a higher recurrence rate. One study found that the marked effect of corticosteroid injections continued until 8 weeks and then began to deteriorate.^[13]^ The greatest pain reduction was achieved in patients treated with corticosteroids 4 weeks after initial treatment.^[12,16]^

The limitations of this systematic review involve the uniformity of the administration program and the small size of the included RCTs. Trials with a high or unclear risk of bias could lower the quality of evidence obtained in our study.^[37]^ There is no general consensus on follow-up time points and the unavoidably arbitrary VAS and DASH scale endpoints used to define “success” in pain reduction and functional improvement, resulting in great heterogeneity among the studies. Regarding PRP injection, standardized preparation, optimal quantity, indications and injection time were originally controversial. More substantiated clinical data about PRP are needed. Consequently, caution should be taken when the estimates obtained from this meta-analysis are interpreted.

## 5 Conclusions

Local corticosteroid injections demonstrated favorable outcome (DASH score) compared with those of local PRP treatments for lateral elbow epicondylitis during the short-term follow-up period (4 weeks and 8 weeks post-treatment). Otherwise, at the long-term follow-up (24 weeks post-treatment), PRP injections had improved pain and function (VAS and DASH score) more effectively than corticosteroid injections. However, higher power studies with much larger sample sizes are advised to obtain more concrete conclusions.

## Author contributions

**Conceptualization:** Ang Li.

**Funding acquisition:** Yanzheng Gao.

**Methodology:** Hongbo Wang.

**Project administration:** Guangquan Zhang.

**Software:** Hongbo Wang.

**Writing – original draft:** Ang Li, Hongbo Wang.

**Writing – review & editing:** Yanzheng Gao, Zhenghong Yu.

Yanzheng Gao orcid: 0000-0001-9474-4710.
